# Development of a Drug Delivery System Using a Compound Based on Ethyl Cyanoacrylate and *Hancornia speciosa* (Gomes) in a Rat Calvaria Model

**DOI:** 10.3390/ph18111695

**Published:** 2025-11-08

**Authors:** Daniel Felipe Fernandes Paiva, Marco Antonio Tridapalli Mafra, Victor Augusto Benedicto dos Santos, Sidney Raimundo Figueroba, Anne Caroline Gercina Carvalho Dantas, Klinger de Souza Amorim, Francisco Haiter Neto, Camila Batista da Silva, Francisco Carlos Groppo

**Affiliations:** 1 Department of Bioscience, Universidade Estadual de Campinas, Piracicaba 13414-903, Brazil; d265738@dac.unicamp.br (D.F.F.P.);; 2 Department of Oral Diagnosis, Universidade Estadual de Campinas, Piracicaba 13414-903, Brazil

**Keywords:** bone regeneration, *Apocynaceae*, *Cyanoacrylates*, models, animal

## Abstract

**Background/Objectives**: Regenerating critical-sized bone defects is a significant clinical challenge. Autogenous bone grafts are the gold standard but have limitations, including donor site morbidity. As an alternative, this study introduces a novel biocomposite combining an ethyl cyanoacrylate (ECA) polymer with *Hancornia speciosa* (Hs) latex. The ECA acts as a scaffold and delivery vehicle for the latex, which contains phytochemicals with known angiogenic properties. **Methods**: We created 5 mm critical-sized calvarial defects in 36 Wistar rats, which were divided into four experimental groups. Bone regeneration was evaluated at 30, 60, and 90 days using micro-computed tomography (micro-CT) for morphometric analysis and hematoxylin and eosin staining for histology. **Results**: The composite-treated group (Hs+ECA) showed significantly higher bone volume (57.2; IQR: 56.7–61.2) than the control (53.9; IQR: 49.4–56.4) and ECA-only (48.4; IQR: 47.2–59.9) groups at 90 days (*p* < 0.05). By day 60, the bone volume in the Hs+ECA group was statistically similar (*p* > 0.05) to that of the autogenous bone group. Histological analysis revealed an organized repair process with neoangiogenesis observed only in the Hs+ECA group, confirming the material’s strong bioactivity. **Conclusions**: The Hs+ECA composite is a promising biomaterial that acts as an effective delivery system for the bioactive components of the latex. The induced angiogenesis was critical to its regenerative success. This cost-effective material warrants further investigation for clinical applications in regenerative dentistry.

## 1. Introduction

The regeneration of critical-sized bone defects resulting from trauma or tumor resections represents a significant clinical challenge that often exceeds the tissue’s endogenous repair capacity, necessitating reconstructive interventions to restore structural integrity [1,2]. Autogenous bone grafting remains the gold standard procedure due to its osteoconductive and osteoinductive properties [1]. However, its clinical use is limited by donor site morbidity and restricted tissue availability [3]. Alternative approaches, including allografts and xenografts, partially address these limitations but introduce complications such as immune responses and reduced biological activity [2].

The constraints associated with traditional grafts have driven the development of synthetic biomaterials that function not merely as passive scaffolds but also as sophisticated drug delivery systems (DDS) [2,3]. The local administration of bioactive molecules faces major challenges due to their short half-lives and rapid clearance from injury sites, often requiring supraphysiological doses that carry inherent risks of adverse effects [2]. An optimal DDS based on biodegradable polymers should protect bioactive molecules, provide sustained and controlled release, and undergo gradual replacement by newly formed bone tissue [3].

Latex derived from *Hancornia speciosa* Gomes (Hs) has emerged as a promising source of bioactive compounds. Its angiogenic and anti-inflammatory activities are attributed to phytochemicals present in the serum fraction—particularly polyphenols such as chlorogenic acid—which have been shown to modulate extracellular matrix remodeling [4]. However, the fluid nature of the latex lacks sufficient stability to remain localized at defect sites, requiring a carrier vehicle capable of controlled and targeted delivery [2,3,4,5].

Ethyl cyanoacrylate (ECA) is a monomer that undergoes rapid anionic polymerization upon exposure to moisture, forming a stable polymeric matrix. This property allows it to function as an effective biological adhesive with well-documented biocompatibility and antimicrobial characteristics [6,7].

The combination of ECA and Hs latex has not been previously reported in the literature. The theoretical basis for this novel combination lies in the hypothesis of functional synergy: ethyl cyanoacrylate would act as a stable osteoconductive matrix whose polymerization process physically encapsulates latex components, enabling a non-covalent release mechanism [2]. Concurrently, the latex-derived phytochemicals would provide bioactive stimulation, primarily enhancing angiogenesis to support graft vascularization [5] and modulating inflammatory responses to establish a pro-regenerative microenvironment [8].

Therefore, this study aimed to evaluate the effects of a mixture of ethyl cyanoacrylate and *Hancornia speciosa* latex on bone regeneration in critical-sized calvarial defects in rats.

## 2. Results

Of the 36 animals initially enrolled, two (n = 2) died during anesthetic induction prior to surgical intervention. Both belonged to the 90-day evaluation group and were therefore excluded from the analysis. The remaining 34 animals completed the experimental protocol, and their data were included in the final analyses.

### 2.1. Micro-Computed Tomography Analysis

Bone parameters obtained from micro-computed tomography did not meet the assumptions of normality or homoscedasticity. Table 1 summarizes the median values and interquartile ranges (25–75%), along with statistically significant intergroup differences.

**Figure 1:**
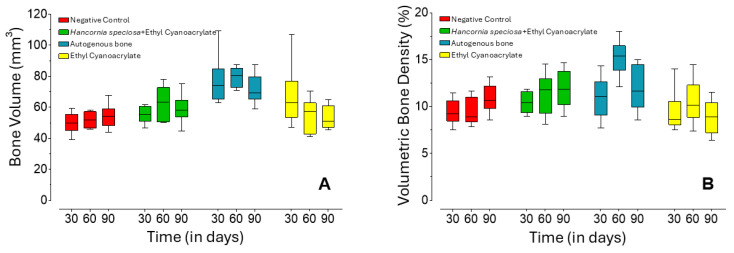
Bone volume (**A**) and bone density (**B**) as a function of the observed treatments and time points. Center line = median; box = 1st and 3rd quartiles; whiskers = maximum and minimum values.

Bone volume (Figure 1A) remained statistically consistent across the three experimental time points (*p* > 0.05) within each treatment group. When analyzed by time point, the autogenous bone (AB) group exhibited significantly greater bone volume at 30 days compared with both the control (*p* = 0.0007) and Hs+ECA (*p* = 0.0111) groups, with no significant difference relative to ECA (*p* = 0.16). At 60 days, bone volume was comparable between AB and Hs+ECA groups (*p* = 0.061); however, AB remained significantly higher than both control (*p* = 0.0003) and ECA (*p* = 0.0023). By 90 days, the AB group continued to display superior bone volume compared with control (*p* = 0.0107) and ECA (*p* = 0.0078) groups.

Bone density (Figure 1B) exhibited temporal variation exclusively within the AB group, which showed significantly higher density at 60 days compared with 30 days (*p* = 0.0205). No other group demonstrated chronological differences. At 60 days, the AB group presented significantly greater bone density than all other treatments. At 90 days, the ECA group exhibited lower density compared with both AB (*p* = 0.0298) and Hs+ECA (*p* = 0.0173) groups.

**Figure 2:**
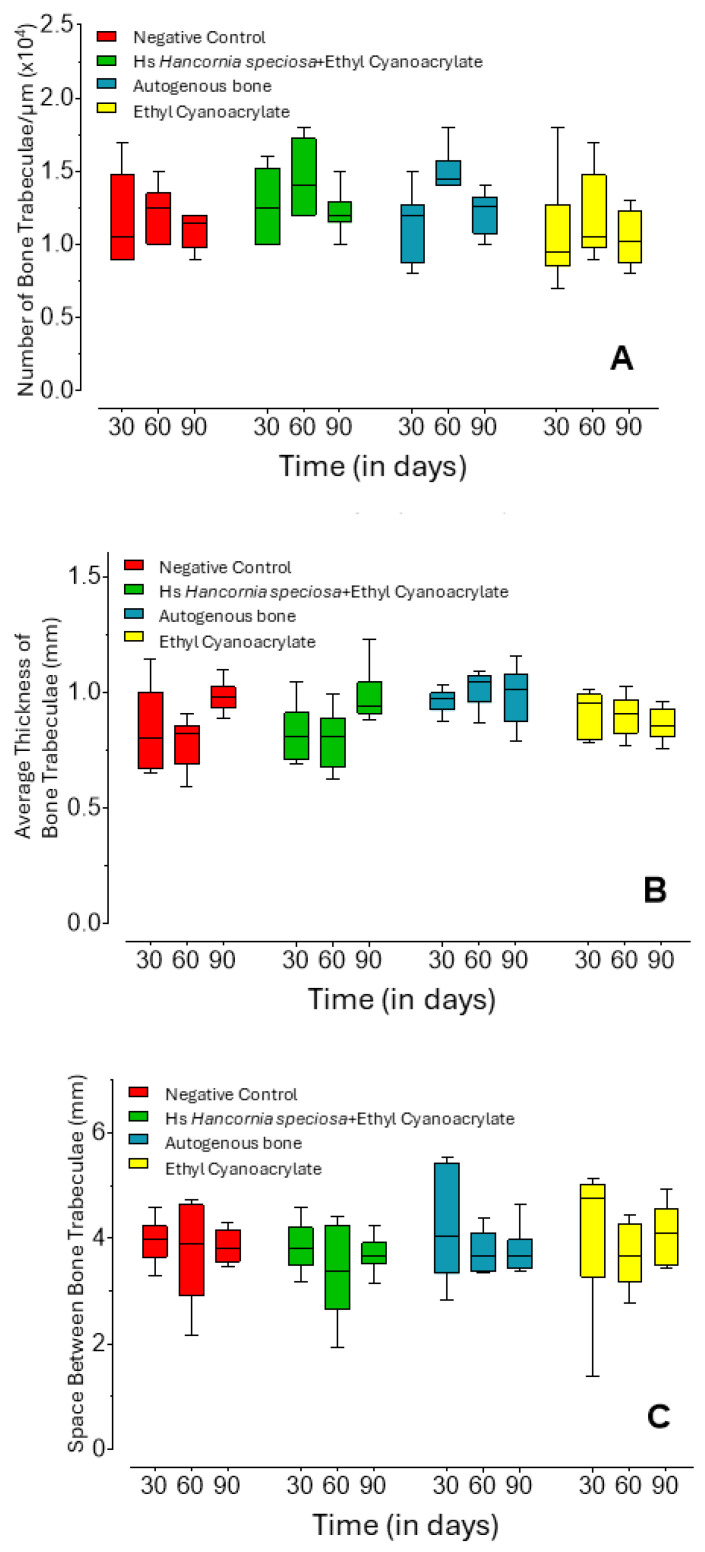
Bone trabecular parameters as a function of the observed treatments and time points. Trabecular number per µm (**A**), mean trabecular thickness (**B**), and trabecular separation (**C**). Center line = median; box = 1st and 3rd quartiles; whiskers = maximum and minimum values.

Trabecular number (Figure 2A) remained generally stable across groups and time points, except in the AB group, which exhibited a significant increase at 60 days compared with both 30 days (*p* = 0.0133) and the ECA group at the same time point (*p* = 0.0210).

Mean trabecular thickness (Figure 2B) showed distinct temporal trends within specific groups. The control group displayed progressive thickening at 90 days relative to 30-day (*p* = 0.0398) and 60-day (*p* = 0.0057) measurements. Similarly, the Hs+ECA group demonstrated increased trabecular thickness at 90 days compared with 60 days (*p* = 0.0401). The AB group consistently maintained higher trabecular thickness than the Hs+ECA group at 30 (*p* = 0.0412) and 60 days (*p* = 0.0024), and exceeded the control group at 60 days (*p* = 0.0009). Trabecular separation (Figure 2C) remained statistically similar across all groups and time points (*p* = 0.7371).

### 2.2. Qualitative Histological Analysis

Following hematoxylin and eosin staining, histological sections were independently assessed by two blinded observers for bone-related parameters. Samples from the same treatment group and time point demonstrated consistent microscopic features.

Untreated Control Group: Sections revealed disorganized connective tissue proliferation without evidence of new bone formation. Inflammatory cell infiltration was prominent at 30 and 60 days but subsided by 90 days. Fibrous capsule formation within the defect was a consistent finding.

*Hancornia speciosa* + ethyl Cyanoacrylate (Hs+ECA) Group: Bone neoformation was evident at the defect margins, accompanied by progressive neovascularization that became more pronounced at 90 days. Inflammatory infiltration, initially marked at 30 days, decreased substantially in later time points.

Autogenous Bone (AB) Group: Defect margins remained well-defined with fibrous tissue invagination throughout the study period. Bone spicule formation bridging graft margins was evident at 60 and 90 days, confirming active bone remodeling.

Ethyl Cyanoacrylate (ECA) Group: Material encapsulation persisted across all time points, with progressive degradation observed by 90 days. The adhesive acted as a physical barrier, preventing disorganized connective tissue invasion into the defect. Bone formation became apparent at 60 days and markedly increased by 90 days. Inflammatory responses, initially intense at 30 days, declined progressively thereafter.

**Figure 3:**
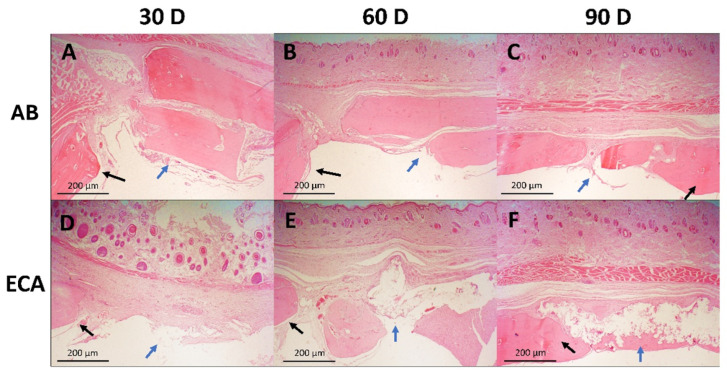
Representative images of the morphology of calvaria subjected to different treatments (n = 6), hematoxylin-eosin staining, 2.5× magnification, scale bar = 200 μm. AB group: 30 days, (**A**): blue arrow shows autogenous bone with initial encapsulation and the presence of inflammatory tissue; black arrow shows unfilled space between the edge of the bone defect and the treatment. 60 days, (**B**): blue arrow shows incomplete encapsulation, but with fibrous tissue already adhered to the autogenous bone; black arrow shows bone tissue in contact with fibrous tissue due to the treatment. 90 days, (**C**): blue arrow indicates infiltrated fibrous tissue intimately adhered to the bone tissue (black arrow). ECA group: 30 days, (**D**): blue arrow indicates the beginning of treatment encapsulation, with little fibrous tissue, and black arrow indicates bone tissue not yet in contact with fibrous tissue. 60 days, (**E**): blue arrow indicates advanced encapsulation of the treatment and the presence of inflammatory tissue; black arrow indicates the beginning of contact between bone tissue and the encapsulating fibrous tissue. 90 days, (**F**): blue arrow indicates fully encapsulated and organized treatment, and black arrows show intimate contact with the bone tissue with the encapsulation fibers.

**Figure 4:**
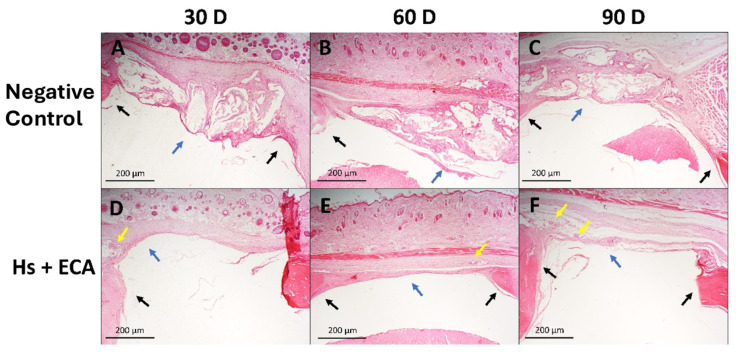
Representative histological images of calvarial defects subjected to different treatments, stained with hematoxylin and eosin. Black arrows indicate the host bone margins of the defect. Magnification: 2.5^X^; scale bar = 200 µm. Control Group: 30 days, (**A**): a fibrous capsule containing inflammatory tissue is observed filling the defect (blue arrow). Control Group: 60 days, (**B**): the fibrous capsule shows tissue undergoing differentiation and remodeling (blue arrow). Control Group: 90 days, (**C**): a more organized and remodeled fibrous capsule persists within the defect (blue arrow). Hs+ECA group: 30 days, (**D**): suggestive evidence of neoangiogenesis (yellow arrow) is observed adjacent to the implanted biomaterial (blue arrow). Hs+ECA group: 60 days, (**E**): evidence of continued angiogenesis is visible near the defect margin (yellow arrow), with initial bone repair processes starting at the defect interface (blue arrow). Hs+ECA group: 90 days, (**F**): suggestive evidence of high neoangiogenesis is present (yellow arrows), with initial bone repair processes starting at the defect interface (blue arrow).

Collectively, these findings are depicted in Figure 3 and Figure 4, illustrating the temporal progression of each treatment modality.

## 3. Discussion

The rat calvarial bone defect model is a well-established preclinical tool for evaluating biomaterials, as its repair process—intramembranous ossification—closely mimics craniofacial bone regeneration [9,10]. The use of a critical-sized defect, which does not heal spontaneously, ensures that any observed bone formation is attributable to the material’s activity [11,12]. This repair process is complex, involving a cascade of inflammation, cell proliferation, and remodeling, making the model particularly suitable for studying implant–host interactions [1,13,14].

In this study, the materials were not subjected to conventional sterilization to avoid altering their physicochemical and biological properties. Ethyl cyanoacrylate is a moisture-reactive monomer, and sterilization methods involving heat or steam could induce premature polymerization [15]. Likewise, the bioactivity of *Hancornia speciosa* latex depends on its complex phytochemical composition, including heat-sensitive proteins and polyphenols [4,16], which could be degraded by thermal sterilization. This rationale is consistent with the literature, where cold methods such as membrane filtration or gamma irradiation are preferred for latex-based materials [5,17]. Despite the absence of sterilization, no signs of infection or rejection were observed in any specimen.

Autogenous bone reaffirmed its superiority as the gold standard [1]. At 30 days, it exhibited greater bone volume than the Control and Hs+ECA groups, and this superiority persisted at 60 days compared with the Control and ECA groups. Notably, the autogenous graft reached a maturation peak at 60 days, demonstrated by a significant increase in both bone density and trabecular number relative to 30 days. At this time point, the AB group presented higher bone density than all other groups and greater trabecular thickness than the Control and Hs+ECA groups. However, graft efficacy depends on mechanical stability; inadequate fixation can cause micromovement and impair new bone formation [10,18], a limitation that may have influenced these results.

Ethyl cyanoacrylate alone acted as an osteoconductive scaffold by maintaining space for repair and serving as a physical barrier. However, its biologically inert nature led to limited regenerative performance and fibrous encapsulation. This limitation was corroborated by quantitative data, which showed lower bone volume and density at later time points compared with the AB and Hs+ECA groups. The ECA group also exhibited a reduced trabecular number relative to the AB group at 60 days. These findings confirm that the polymer scaffold alone lacks osteoinductive capacity [19,20].

The Hs+ECA composite performed significantly better than ECA alone, with bone volume approaching that of autogenous bone at 60 days. This performance is also promising when compared with other alloplastic biomaterials tested in similar models, such as PCL–hydroxyapatite membranes [21]. The most notable histological finding in this group was the presence of neoangiogenesis, a key event for the regeneration of critical-sized defects [1]. The observation of blood vessels supports the hypothesis that the latex exerted its bioactive function, attributed to phytochemicals such as chlorogenic acid [4]. The success of this localized delivery underscores the importance of site-specific application, as systemic (oral) administration of the same latex has been shown to be ineffective for bone neoformation [22]. Moreover, the materials displayed distinct healing dynamics: while the autogenous graft peaked at 60 days, the Hs+ECA group exhibited ongoing remodeling at later stages. This was evidenced by the significant increase in trabecular thickness between 60 and 90 days (*p* = 0.0401), suggesting a slower but continuous regenerative process.

These results support the interpretation of the Hs+ECA composite as a drug delivery system [3]. In this model, the ECA polymer serves as the vehicle, while the bioactive latex components act as the pharmacological agent [2]. The superior performance of the Hs+ECA group compared with the ECA-only group provides direct evidence of successful delivery. The proposed mechanism involves non-covalent physical encapsulation, in which the rapid polymerization of ECA traps latex components, allowing their sustained release [2]. The observed neoangiogenesis provides functional confirmation that the “drug” was released and remained biologically active, leading to bone regeneration superior to that achieved with the inert vehicle alone. This study therefore validates the use of ethyl cyanoacrylate as a DDS platform for delivering fluid bioactive agents.

This study presents some limitations that must be considered. The lack of sterilization, although a methodological decision to preserve bioactivity, could have introduced bacterial contamination. Additionally, the absence of detailed physicochemical characterization of the latex and the final composite affects reproducibility, as does the lack of rigid graft fixation. The reliance on qualitative histology further highlights the need for future investigations to incorporate quantitative methods. Future studies should address these limitations by employing robust fixation techniques and including quantitative analyses, such as histomorphometry and immunohistochemistry, to provide more definitive data. Despite these constraints, the present study confirms the potential of the composite and establishes a clear direction for future research.

## 4. Materials and Methods

### 4.1. Ethical and Legal Considerations

This study was approved by the Animal Use Ethics Committee (CEUA) of the Institute of Biology at the State University of Campinas (UNICAMP) under protocol number 5700-1/2021. The use of animal models was essential to replicate controlled physiological conditions and to evaluate biomaterial efficacy in accordance with the ARRIVE guidelines. The study protocol was developed a priori and submitted to the ethics committee, including all methods and planned outcomes; however, it was not registered in a public database before the start of the experiments.

### 4.2. Sample Size and Animals

Sample size was determined using a 95% confidence interval and a standard error of ±5% of the mean (50%), resulting in six animals per experimental group. Thirty-six male rats (HanUnib: WH Wistar, SPF), aged 10–12 weeks and weighing 400–450 g, were obtained from CEMIB-UNICAMP. Animals were housed in standard plastic cages under controlled environmental conditions: 12 h light/dark cycles, temperature maintained at 22 ± 2 °C, controlled humidity, and ad libitum access to balanced feed and water.

Animals were randomized into four experimental groups using a randomization table generated in Microsoft Excel. Allocation to the euthanasia time points (30, 60, or 90 days) was based on body weight on the day of the experiment, with the heaviest animals selected to minimize the risk of anesthetic overdose. To reduce potential sources of bias, all procedures were performed by the same operator using a surgical loupe, and the cage positions within the animal facility were rotated twice weekly as part of the cleaning routine.

### 4.3. Materials

The experimental materials included ethyl cyanoacrylate adhesive (Super Bonder^®^, Henkel Ltd.a., Jundiaí, Brazil) and 50% *Hancornia speciosa* latex (Rei da Mangaba^®^, Serro, Brazil). Precision measurements were performed using P200 and P1000 pipettes (Gilson, Villiers-le-Bel, France). Anesthetic agents consisted of ketamine hydrochloride and xylazine (Vetbrands, São Paulo, Brazil). Surgical instruments included a 5 mm bone trephine (Dentoflex, São Paulo, Brazil) and 5-0 nylon sutures (Techsuture, Bauru, Brazil).

### 4.4. Biomaterial Formulation

The Hs+ECA formulation was prepared by combining 0.2 mL of ethyl cyanoacrylate with 1 mL of 50% *Hancornia speciosa* latex, mixed until homogeneous in a sterile container, and dried at 31.5 ± 2 °C for 30 min. After dehydration, the material was ground into granules and precisely weighed into mini-Eppendorf tubes (0.0180 g per tube) to fit 5 mm defects. Samples were stored at 5 ± 2 °C, protected from light with aluminum foil wrapping. Figure 1 shows the materials used for biomaterial processing and the final appearance of the weighed product.

The ECA group received ethyl cyanoacrylate applied directly to the bone defects, where polymerization occurred upon contact with the blood clot. Positive controls were treated with autogenous bone grafts harvested from the contralateral hemisphere, while negative controls received only blood clot formation.

### 4.5. Surgical Protocol

Critical-sized (5 mm) calvarial bone defects were created following the protocol adapted from Sánchez-Garcés et al. (2020) [23]. All animals corresponding to each time point were operated on the same day. Anesthesia was induced by intraperitoneal injection of xylazine (10 mg/kg) and ketamine (90 mg/kg), followed by hair removal and antisepsis with 10% povidone–iodine solution.

A 2 cm midline incision was made using a surgical scalpel to expose the parietal bones. Circular defects were prepared with a 5 mm trephine operating at 500 rpm under continuous 0.9% saline irrigation. Each animal received two distinct treatments for subsequent evaluation by microtomography and histological analysis. Postoperative monitoring included assessment using the Grimace Scale, along with weekly evaluations of body weight and food and water intake. Euthanasia was performed at predetermined intervals (30, 60, and 90 days) by anesthetic overdose.

Humane endpoints were established: animals were monitored daily, and euthanasia would be performed in cases of weight loss exceeding 20% of baseline on the day of surgery, or in the presence of infection or poor wound healing unresponsive to treatment. No animals met the humane endpoint criteria before the scheduled study endpoints.

### 4.6. Micro-Computed Tomography Analysis

The primary outcome variable was bone volume (mm^3^). Cranial specimens were scanned using a SkyScan 1174 micro-CT (Kontich, Bélgica) system under the following parameters: 50 kVp, 800 μA, 0.5 mm aluminum filter, 30.04 μm voxel resolution, and 180° rotation generating 360 projections per specimen. Image reconstruction was performed using NRecon software (v. 1.7.3.0), and three-dimensional alignment was achieved with DataViewer. Volumetric analyses were carried out using SkyScan CT Analyser software (v. 1.17.7.2+) to quantify bone volume, volumetric density, trabecular thickness, trabecular separation, and trabecular number.

### 4.7. Histological Preparation

Samples were fixed for 24 h at 4 °C in 10% formaldehyde in phosphate buffer (pH 7.4) for histological examination. Decalcification was performed for 90 days in a 7% EDTA solution in phosphate buffer (pH 7.4) with daily solution changes. After decalcification, samples were dehydrated, cleared, and embedded in Paraplast. Coronal sections, 6 µm thick, were obtained using a manual microtome and stained with hematoxylin and eosin.

### 4.8. Morphometric Analysis

Histological images were captured using a Nikon photomicroscope Eclipse E800 (Tokyo, Japan) equipped with a Nikon FDX-35 (Tokyo, Japan) imaging system. Morphometric evaluation was performed by two independent, blinded observers who qualitatively assessed and documented tissue characteristics. Observers analyzed bone formation components, signs of inflammation, and the presence of blood vessels.

### 4.9. Statistical Analysis

Microtomographic data were compiled in Microsoft Excel and analyzed using GraphPad Prism 8.0. Statistical evaluation included Bartlett’s test for homoscedasticity, Shapiro–Wilk test for normality, Kruskal–Wallis test for group comparisons, and Dunn’s post hoc test for multiple pairwise comparisons. Statistical significance was set at α = 0.05.

## 5. Conclusions

The combination of ethyl cyanoacrylate and *Hancornia speciosa* latex effectively promoted bone regeneration in critical-sized calvarial defects in rats. Morphometric analysis revealed that the composite formulation (Hs+ECA) demonstrated superior performance compared to the polymer alone (ECA). While initially showing inferior results relative to autogenous grafting, the composite exhibited progressive tissue maturation, encouraging regenerative potential in bone volume parameters by 60 days, but still lower than autogenous bone. Histopathological examination revealed that the most significant finding was neoangiogenesis induction within the composite-treated group—a distinctive bioactive characteristic absent in other treatment modalities. This response indicates favorable and well-organized tissue regeneration processes.

These findings establish the composite as a functionally bioactive material wherein ethyl cyanoacrylate serves as an efficient delivery vehicle for latex-derived phytochemicals. The pro-angiogenic properties of these bioactive compounds proved essential to the observed regenerative outcomes. This preclinical investigation provides compelling proof of concept for a cost-effective biomaterial approach. Nevertheless, additional research is warranted to optimize the formulation, establish appropriate sterilization protocols, and assess performance in load-bearing long bone applications prior to clinical translation.

## Figures and Tables

**Table 1:** Bone microstructural parameters assessed by micro-computed tomography for the different groups and time points.

	Group	30 Days (n = 12)	60 Days (n = 12)	90 Days (n = 10)
Bone Volume (mm^3^)	Hs+ECA	55.6 ^b A^ (53.0–59.3)	63.2 ^b A^ (53.7–69.0)	57.2 ^a A^ (56.7–61.2)
	Negative Control	50.0 ^b A^ (47.0–53.8)	51.8 ^b A^ (47.2–56.5)	53,9 ^b A^ (49.4–56.4)
	AB	73.9 ^a A^ (68.2–76.1)	80.2 ^a A^ (75.1–83.4)	67.3 ^a A^ (67.1–77.2)
	ECA	62.8 ^ab A^ (56.6–66.8)	57.2 ^ab A^ (46.6–59.7)	48.4 ^b A^ (47.2–59.9)
Volumetric Bone Density (%)	Hs+ECA	10.4 ^a A^ (9.6–11.4)	11.8 ^b A^ (10.1–12.4)	11.8 ^a A^ (10.6–13.5)
	Negative Control	9.2 ^a A^ (8.8–10.1)	8.9 ^b A^ (8.6–10.3)	10.5 ^ab A^ (10.2–11.9)
	AB	11.1 ^a A^ (9.8–12.0)	15.4 ^a B^ (14.7–15.9)	11.4 ^a AB^ (10.4–14.3)
	ECA	8.6 ^a A^ (8.3–9.2)	10.2 ^b A^ (9.5–11.3)	8.9 ^b A^ (7.5–10.1)
Trabecular Number (1/µm × 10^−4^)	Hs+ECA	1.25 ^a A^ (1.03–1.48)	1.40 ^ab A^ (1.23–1.65)	1.20 ^a A^ (1.20–1.20)
	Negative Control	1.05 ^a A^ (0.90–1.35)	1.25 ^ab A^(1.05–1.30)	1.20 ^a A^ (1.00–1.20)
	AB	1.20 ^a A^ (0.98–1.20)	1.45 ^a B^ (1.40–1.50)	1.30 ^a AB^ (1.10–1.30)
	ECA	0.95 ^a A^ (0.90–1.08)	1.05 ^b A^ (1.00–1.33)	1.00 ^a A^ (0.90–1.20)
Mean Trabecular Thickness (mm)	Hs+ECA	0.81 ^b AB^ (0.73–0.86)	0.81 ^b A^ (0.72–0.85)	0.94 ^a B^ (0.92–0.95)
	Negative Control	0.80 ^ab A^ (0.71–0.92)	0.82 ^b A^ (0.74–0.84)	0.98 ^a B^ (0.95–1.00)
	AB	0.97 ^a A^ (0.94–0.99)	1.04 ^a A^ (1.00–1.06)	1.03 ^a A^ (0.90–1.05)
	ECA	0.95 ^ab A^ (0.83–0.98)	0.91 ^ab A^ (0.85–0.95)	0.85 ^a A^ (0.83–0.92)
Trabecular Separation (mm)	Hs+ECA	3.80 ^a A^ (3.62–4.03)	3.38 ^a A^ (2.98–4.04)	3.65 ^a A^ (3.64–3.81)
	Negative Control	3.97 ^a A^ (3.80–4.08)	3.90 ^a A^ (3.27–4.50)	3.75 ^a A^ (3.59–4.13)
	AB	4.03 ^a A^ (3.55–5.13)	3.67 ^a A^ (3.44–3.95)	3.59 ^a A^ (3.44–3.73)
	ECA	4.75 ^a A^ (4.05–4.99)	3.67 ^a A^ (3.38–4.08)	4.09 ^a A^ (3.51–4.42)

Values are expressed as median (25–75% interquartile range). Lowercase superscript letters (a, b): Indicate statistical comparisons between groups within the same time point (column). Values in the same column that do not share a common letter are significantly different (*p* < 0.05). Uppercase superscript letters (A, B): Indicate statistical comparisons within the same group over time (row). Values in the same row that do not share a common letter are significantly different (*p* < 0.05). Abbreviations: Hs+ECA: *Hancornia speciosa* and ethyl-cyanoacrylate group; Control: Empty defect (Negative Control); OA: Autogenous bone; ECA: Ethyl-cyanoacrylate.

## Data Availability

The original contributions presented in this study are included in the article. Further inquiries can be directed to the corresponding author.

## Abbreviations

The following abbreviations are used in this manuscript:

Hs	Hancornia *speciosa* (Gomes)
ECA	Ethyl cyanoacrylate
micro-CT	Micro-computed tomography
DDS	Drug delivery systems
AB	Autogenous bone
